# Genetic Structure, Linkage Disequilibrium and Association Mapping of Verticillium Wilt Resistance in Elite Cotton (*Gossypium hirsutum* L.) Germplasm Population

**DOI:** 10.1371/journal.pone.0086308

**Published:** 2014-01-23

**Authors:** Yunlei Zhao, Hongmei Wang, Wei Chen, Yunhai Li

**Affiliations:** State Key Laboratory of Cotton Biology, Institute of Cotton Research of Chinese Academy of Agricultural Sciences (CAAS), Anyang, People's Republic of China; New Mexico State University, United States of America

## Abstract

Understanding the population structure and linkage disequilibrium in an association panel can effectively avoid spurious associations and improve the accuracy in association mapping. In this study, one hundred and fifty eight elite cotton (*Gossypium hirsutum* L.) germplasm from all over the world, which were genotyped with 212 whole genome-wide marker loci and phenotyped with an disease nursery and greenhouse screening method, were assayed for population structure, linkage disequilibrium, and association mapping of Verticillium wilt resistance. A total of 480 alleles ranging from 2 to 4 per locus were identified from all collections. Model-based analysis identified two groups (G1 and G2) and seven subgroups (G1a–c, G2a–d), and differentiation analysis showed that subgroup having a single origin or pedigree was apt to differentiate with those having a mixed origin. Only 8.12% linked marker pairs showed significant LD (P<0.001) in this association panel. The LD level for linked markers is significantly higher than that for unlinked markers, suggesting that physical linkage strongly influences LD in this panel, and LD level was elevated when the panel was classified into groups and subgroups. The LD decay analysis for several chromosomes showed that different chromosomes showed a notable change in LD decay distances for the same gene pool. Based on the disease nursery and greenhouse environment, 42 marker loci associated with Verticillium wilt resistance were identified through association mapping, which widely were distributed among 15 chromosomes. Among which 10 marker loci were found to be consistent with previously identified QTLs and 32 were new unreported marker loci, and QTL clusters for Verticillium wilt resistanc on Chr.16 were also proved in our study, which was consistent with the strong linkage in this chromosome. Our results would contribute to association mapping and supply the marker candidates for marker-assisted selection of Verticillium wilt resistance in cotton.

## Introduction

Cotton is an important economic crop worldwide, which provides the most important natural fiber for the textile industry. Genetic improvement of yield, fiber quality and disease resistance is the most important objectives in cotton breeding programs worldwide. However, it is a challenging task for breeders to realize the synchronous improvement of yield, fiber quality and disease resistance because of the negative genetic correlation between them [1]. The development of molecular quantitative genetics has made it possible to map the quantitative trait loci (QTLs) for yield, fiber quality and disease resistance, thus facilitating the application of marker-assisted selection (MAS) for genetic improvement. In cotton, numerous QTLs for yield, fiber quality and disease resistance were identified [2]–[11]. In all these studies, the QTL mapping had been performed in segregating populations derived from biparental crosses. Due to limited recombination events, it is difficult for biparental segregating populations to detect closely linked markers for marker-assisted selection. What's more, the frequency of polymorphic loci in biparental populations is limited and some minor QTLs are not detected. An alternative approach to QTL mapping is association analysis, also known as LD mapping. In contrast to QTL mapping based on bi-parental populations, association mapping is based on linkage disequilibrium (LD) and uses a sample of lines from the broader breeding population, unrelated by any specific crossing design [12]. So, the higher number of historical recombination events can be explored in natural population than that in the biparental segregating populations, resulting in a higher resolution of QTL mapping [13]. What's more, association mapping has been used to identify causal polymorphism within a gene that is responsible for the phenotypic variations [14].

The starting point for association mapping studies is based on the non-random association of alleles at different loci (linkage disequilibrium, LD), namely between a marker locus and a phenotypic trait locus. LD can be caused by unknown population structure and several forces, including mutation, drift, genetic bottlenecks, founder effects, selection, and specifically for plants, level of inbreeding caused by their mating systems [15]. In order to appropriately apply LD mapping in crop plants, it is a prerequisite to characterize LD levels and patterns in a population analyzed. It is also important to distinguish between physical LD and the other different forces that can create LD in natural populations, to avoid the detection of spurious associations [16]. The decay or decrease of LD with increasing map distance between markers in outcrossing plants is usually faster than that in inbreeding plants [16]. For example, LD decays rapidly within 1–5 kb in maize diverse inbred lines [17], 1.1 kb in cultivated sunflower [18], 300 bp in wild grapevine [19], whereas LD decays slowly within 250 kb in Arabidopsis [20], 212 kb in elite barley cultivars [21], 100–200 kb in rice diverse lines [22], [23] and 250 kb in cultivated soybean [24]. Also, The decay or decrease of LD in wild relatives is faster than that in modern varieties [25], [26].

The tetraploid species, *Gossypium hirsutum* L. (n = 26, AD genome), showing obvious economic importance such as high yield and environmental suitability, have attracted considerable scientific interest for plant breeders and agricultural scientists and been planted widespreadly, and have been responsible for 95% of the annual cotton crop in the world [27]. However, due to the complexity of genome structure and the lack of high-quality molecular markers, studies on the population structure and LD in cotton (*Gossypium hirsutum* L.) is limited so far and lagged behind the other crop species. Recently, several studies have investigated the level of linkage disequilibrium among genetic markers in various cotton populations. For example, at the significance threshold (*r* ^2^≥0.1), LD decays up to 25 cM in 335 *G. hirsutum* germplasm [28], less than 10 cM in 208 landrace stocks and more than 30 cM in 77 photoperiodic variety accessions [29], while LD decays within 13–14 cM in 81 Upland cotton cultivars [30]. The fast LD decay of cotton cultivars illustrates the significant potential for LD-based association mapping for agronomic traits. Elite cotton (*Gossypium hirsutum* L.) germplasm is the important resources in cotton breeding, which possess the following one or more characters of high yield, good fiber quality, earliness, disease and pest resistance. Therefore, further characterization of the population structure and LD levels in elite cotton (*Gossypium hirsutum* L.) germplasm collected from all over the world will be a benefit for association mapping of complex traits in cotton.

Verticillium wilt, incited by fungal pathogen called *Verticillium dahliae*, is a serious soil-borne disease with international consequences for cotton production. To date, the most effective and feasible way to control wilt disease has been to develop new cotton varieties resistant to Verticillium wilt. Since most commercial cultivars of upland cotton are susceptible or only slightly resistant to cotton wilt disease [10], it is necessary for cotton breeder to improve Verticillium wilt resistance in cotton (*Gossypium hirsutum* L.) by conducting introgression of resistance genes in sea island cotton or gene pyramiding from different sources of resistance. The most effective selection for introgression of resistance genes or gene pyramiding was using marker-assisted selection (MAS). To date, at least 60 different Verticillium wilt resistance QTLs have been reported on 10 chromosomes or linkage groups of cotton [8]–[11]. However, these QTLs were mapped in four different bi-parental populations and poorly colocalized, thus markers linked to these QTLs are not directly used in cotton breeding. QTL effects needed to be verified in other genetic backgrounds prior to widespread application of QTL-linked markers in MAS. Against this backdrop, association mapping using a sample of lines from the broader breeding population showed great potential for QTL detection, which can explore the higher number of historical recombination events than the biparental segregating populations, resulting in a higher resolution of QTL mapping. Currently, association mapping for yield and fiber traits in cotton has been conducted in several studies [28]–[30], but no report has been found for association mapping of Verticillium wilt resistance in cotton. Therefore, our study on association mapping of Verticillium wilt resistance in cotton would be a beneficial supplementary and verification for current QTL mapping of verticillium wilt resistance genes in cotton.

In this study, we genotyped a population of 158 elite cotton (*Gossypium hirsutum* L.) germplasm from all over the world using genome-wide molecular markers. The aims of this study were to assess the population structure, linkage disequilibrium (LD), and association of molecular markers with Verticillium wilt resistance in a collection of 158 elite cotton germplasm accessions.

## Materials and Methods

### Sampling of cotton accessions

A collection of 329 cotton (*Gossypium hirsutum* L.) accessions from the China cotton germplasm collection were planted in the experimental field at Cotton Research Institute of Chinese Academy of Agricultural Sciences, Anyang, China in 2007,2008 and 2009 to evaluate their agronomic traits of yields, fiber quality, growth period and disease resistance. Some of varieties with same pedigree and similar performance in agronomy traits were excluded. Finally, a panel of 158 cotton accessions () were selected, which included 106 accessions from China, 41 accessions from America, 3 accessions from Africa, 4 accessions from Former Soviet Union, 1 accession from French, 1 accession from Pakistan, 1 accession from Australia, and 1 accession with unknown origin. These accessions have been strictly self-pollinated during the past decades for germplasm renewing and the residual heterozygosity have been decreased remarkably.

### Phenotypic evaluation

Verticillium wilt resistance of 158 cotton lines was evaluated by two methods. *Verticillium dahliae* isolate Vd080, which is a defolianting, moderate pathogenic to cotton, was used in both methods. The resistance during adult-plant stage was evaluated in the artificial Verticillium wilt nursery in Anyang, China, in 2009. The disease nursery was constructed by unifomly mixing natural soil with *Verticillium dahliae* cotton seed cultivation with an amount of 450∼750 kilogram per hectare. To ensure enough and uniform infection, the disease nursery was devided into several disease pools and each pool was isolated physically from natural soil around it and below. The inoculation amount for each pool was controlled artificially to ensure the uniform and severe infection. At the same time, for each pool, the same susceptible control was used to judge the severity of infection and decrease the error of investigation in different pools (see below). Based on the experiences for many years, the disease pools in the nursery had been severly infected with *Verticillium dahliae*, which ensured the susceptible control to reach anticipative severity of infection (see below). The experimental design was a randomized block with three replications. The different cotton lines were grown in two row plots, 6.0 m long and 0.8 m row space. Jimian 11 acted as a susceptible control to estimate the severity of disease and determine the optimal time for investigation. The 0–5 scales were used for disease severity ratings based on the percentage of diseased leaves of the whole plants, where 0  =  no symptoms, 1  =  less than 25%, 2 = 25–50%, 3 = 50–75%, and 4  =  more than 75% of leaves showing symptoms. Since the 0–5 scales were based on the investigation for a single plant and were not visual to estimate the severity of infection for a certain line, they were converted into the disease index (DI). The disease index (DI) was calculated as follows: 
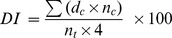
, where *d_c_* was disease severity rating, *n_c_* the numbers of plants with each of the corresponding disease severity rating, and *n_i_* the total number of plants assessed. Since the disease nursery was composed of several disease pools and each pool has the same susceptible control, the DI was further adjusted into relative disease index (RDI) to decrease the error of investigation in different pools by a correction factor K. The K was defined as follow: 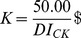, where 50.00 was regarded as the standard DI of the susceptible control, which means the uniform and severe infection in the pool; 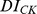 was the factual DI of the susceptible control. The RDI was defined as follow: 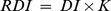, where *DI* was the factual DI of the testing lines. The cotton seeds were planted at the end of April, and the plants usually showed symptoms of Verticillium wilt in June. The disease symptoms gradually increased along with the growth of cotton, and reached their peak in blossoming and boll forming stage. After disease symptoms appeared, all the cotton lines were observed for the severity of infection. The disease severity ratings of the susceptible control were investigated at regular intervals. When the DI of the susceptible control was around 50 (optimally in the range of 40 to 66.67), implying the uniform and severe infection, the disease severity ratings of each cotton line was investigated and the disease index (DI) and relative disease index (RDI) were calculated, which served as an evaluation of disease resistance for a cotton line at adult-plant stage.

The second method was to estimate disease resistance at seedling stage. The experiment was conducted in a greenhouse with 12-h photoperiod and the temperature variation of 23–30°C. The experimental design was also a randomized block with three replications. Cotton seeds were sown in paper pots (6 cm in diameter and 10 cm in height, made up of newspaper and without bottom) filled with autoclaved substrate (vol/vol, vermiculite:sand  =  6∶4). The paper pots were placed on plastic trays. *Verticillium dahliae* Vd080 was cultured in Czapek liquid medium for 10 days. Spores were collected by filteration with 4 lays gauzes and diluted by sterilized distilled water to approximately 1.0×10^7^ spores/ml. Seedlings were inoculated with spore suspension 18 or 22 days after sowing. The seedlings were inoculated by placing the paper pots onto a plate (10 cm in diameter) containing 20 ml of a spore suspension and incubating for 40 min; the pots were then returned to the plastic trays. Seedlings dipped in sterile water were used as the control. Each treatment with three replications(n = 3) had five pots, and each pot contained three to five plants. The susceptible line-Jimian 11 acted as a susceptible control for estimation of RDI. The cotton seedlings generally showed up symptoms 7 days after inoculation. At 18 and 25 days after inoculation, the level of severity was recorded. The disease rating scale was as follows: 0  =  healthy plants, no symptoms on leaves; 1  =  one or two cotyledons showing symptoms and no symptoms on true leaves; 2  =  both cotyledons and one true leaf showing symptoms; 3  =  both cotyledons and two true leaves showing symptoms; 4  =  all of the leaves showing symptoms, symptomatic leaves dropped, the apical meristem was necrotic or the plant died. The disease index (DI) and relative disease index (RDI) were calculated according to the same method as those for the disease nursery in the field above.

### SSR genotyping

Genomic DNA was isolated individually from each of the 158 cotton accessions, starting from fresh leaf tissues and using the CTAB method [31]. A total of 1482 SSRs on the AD-genome wide Reference Map (http://www.cottongen.org/tools/cmap/viewer), which evenly distributed on 26 chromosomes in cotton, were screened and those SSRs showing polymorphism among the 158 cotton accessions were retained for genotyping. Another 17 SSRs and 2 RGAP markers showing polymorphism but without chromosome location information were also included in the analysis (Table S2).Since not all the SSRs on the AD-genome wide Reference Map showed polymorphism among the 158 cotton accessions, we only got 212 informative markers which were used for genotyping. The chromosome locations of these SSR markers and positions of each locus(Table S2)were obtained from the AD-genome wide Reference Map (http://www.cottongen.org/tools/cmap/viewer) and previous studies [32]–[41]. For each marker, only the clear major bands were recorded and each band was corresponding to an allele. Each SSR locus was scored with “1” for one band, “2” for another band, and “3” for the third band, etc., which distinguished different alleles. The occasional non-amplification or missing data state was scored with “−9” or “?”, depending on the software requirement. To avoid assigning incorrect allelic relationships,the following criteria were used: (i) alleles were regarded as belonging to the same locus if they showed an obvious codominant relationship from their segregation patterns among different lines; (ii) when amplicons of the alleles were very close but different in molecular size, they were considered allelic; and (iii) ampicons that were not judged their allelic relationship with the above criteria were regarded as novel loci. Most SSRs were considered codominant markers. For dominant SSRs showing only one band in some lines and no band in other lines, the present state was regarded as a allele and scored with “1”, and the absent state as another allele and scored with “2”. Markers were analysed by PCR and 6% polyacrylamide gel electrophoresis (PAGE). PCR runs were performed 35 cycles of 45 s at 94_°C, at the annealing temperature for 45 s and 72_°C for 90 s, and a final extension step at 72_°C for 10 min. For each SSR locus, alleles were scored in ascending order according to the amplified fragment size.

### Statistical analysis

#### Genetic diversity

The cotton germplasm used in this study were strictly self-pollinated during the past decades for germplasm renewing. Preliminary analysis conducted on this panel using 83 simple sequence repeats (SSR) markers (not shown) showed only a couple of heterozygous loci in a few individuals. Therefore, the individuals in the present study were assumed to be homozygous. The number of alleles, gene diversity, and polymorphism information content (PIC) were estimated using the PowerMarker version 3.25 [42]. The differences of allele richness between different samples were compared using the rarefraction method in the HP-RARE package [43]. The significance of different statistics including gene diversity, PIC and allelic richness was assessed using Wilcoxon's paired test across loci.

#### Population structure and differentiation analyses

The model-based (Bayesian) cluster software STRUCTURE 2.2 [44] was chosen to estimate the population structure of the 158 cotton accessions and assign accessions to groups or subgroups with the 212 molecular markers which distributed across all cotton chromosomes. For structure analysis, each individual was coded using a two-row format: (*x_j_^i, 1^*, *x_j_^i, 2^*), which represents the genotype of individual i at locus j as described by Pritchard et al. [44]. We ran STRUCTURE under the ‘admixture model’ with a burn-in period of 10 000 followed by 100 000 replications of Markov Chain Monte Carlo. Five independent runs each were performed with the number of clusters (*K*) varying from 1 to 15. An ad hoc measure *ΔK* based on the relative rate of change in the likelihood of the data between successive *K* values were used to determine the optimal number of clusters [45]. That run with the maximum likelihood was adopted to divide the cotton accessions into different groups with the membership probabilities threshold of 0.60 as well as the maximum membership probability among groups. Those accessions with less than 0.60 membership probabilities were retained in the admixed group. The inferred groups were further subdivided into subgroups using a similar methodology. Because the pedigree information of many cotton accessions was unknown, the classification of the accessions was largely based on the STRUCTURE results. No a priori population information was used.

The unrooted neighbor-joining(N-J) tree was applied using the software Powermarker 3.25 under the Nei 1983 model [42] to investigate the tridimensional structure of elite cotton germplasm accessions. Using inferred groups and subgroups, genetic differentiation within and among predefined groups and pairwise Fst genetic distances were measured by molecular variance analysis (AMOVA) using ARLEQUIN2.0 [46], with 1,000 permutations and sum of squared size differences as molecular distance.

#### Relative kinship

Pairwise kinship estimates were calculated by constructing relative kinship matrix according to Hardy and Vekemans [47] using the software SPAGeDi. The kinship matrix compared the identity by descent (IBD) among all pairs of the 158 cotton accessions genotyped using 212 markers, by adjusting the probability of identity by state between two individuals with the average probability of identity by state between random individuals. All negative kinship values between individuals were set to zero [48].

#### Linkage disequilibrium

LD was estimated by calculating the square value of correlation coefficient (*r* ^2^) between all pairs of markers with the software package TASSEL 2.1[49]. Only marker loci with minor allele frequency values above 0.05 and having at least 80% successful calls among the sample set were included further for LD analyses. P-values for each *r* ^2^ estimate were obtained with a two-sided Fisher's exact test as implemented in TASSEL. Each pair of loci was categorized as unlinked (marker loci located on different chromosomes) or linked (marker loci located on the same chromosome).The LD was estimated for global, linked and unlined markers, respectively. The LD values between all pairs of marker loci were plotted as triangle LD plots using TASSEL to estimate the general view of genome-wide LD patterns and evaluate ‘block-like’ LD structures. To display the change in LD as a function of genetic distance, the position information of linked markers was acquired according to position references (Table S2), and only when the position information of linked markers came from the same position reference was the genetic distance calculated. The *r* ^2^ value corresponding to the genetic distance was acquired by running the software. LD plots against genetic map distance were generated in Microsoft Excel, where only *r* ^2^ values with P<0.001 were included. The *r* ^2^ value for marker distance of 0 cM was assumed to be 1 as previously described [50]. A curve was drawn to describe the trend of LD decay using the nonlinear regression model, which revealed an overall correlation between the genetic distance of markers on the same chromosome and LD.

#### Association analysis

Since most lines in the cotton panel have no or very weak kinship, the general linear model (GLM) was performed to calculate the marker-trait association using the TASSEL 2.1 software package [49]. In GLM model, association was estimated by using the percentages of admixture of each accession (Q matrix) as covariates to take population structure into account, thus avoided the detection of spurious associations. The Q matrix was created by programme STRUCTURE 2.2. The significant associations were compared with published literature information to judge obtained associations. The 5% ‘minor alleles’ filtered SSR datasets were used for this association mapping models.

## Results

### Phenotypic analysis of Verticillium wilt resistance

**Figure 1:**
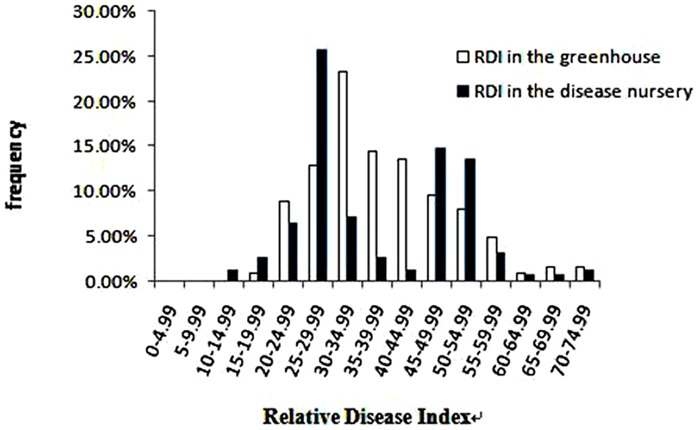
The frequency distribution of Verticillium wilt resistance ratings (indicated by RDI) of 158 accessions in the disease nursery and in the greenhouse environment.

Each line was rated for Verticillium wilt resistance in the disease nursery and in the greenhouse, respectively. The DI was calculated and adjusted into RDI with a correction factor K. The RDI of all the lines ranged from10.10 to 76.60, with an average of 38.23 in the Verticillium wilt nursery environment and from 17.01 to 72.63, with an average of 38.51 in the greenhouse environment, and the coefficient of variation (CV) was 0.34 and 0.29, respectively. ANOVA showed that there were not significant differences for the estimated RDI between two environments (no significant environment effect), but there were significant differences (P<0.001) for RDI among the 158 lines (Table 1). The frequency distribution of RDI showed a continuous variation (Figure 1).

**Table 1:** ANOVA of Verticillium wilt resistance ratings (indicated by RDI) in two environments, Verticillium wilt nursery environment and greenhouse environment.

Source	DF	Sum of squares	Mean square	*F* value	*P* value
Environment	1	37.722	37.722	0.439	0.509
Genotype	157	33243.084	211.739	2.463	<0.0001
Error	157	13499.436	85.984		
Total	315	46780.242			

### Population structure and kinship in the panel of 158 cotton accessions

**Figure 2:**
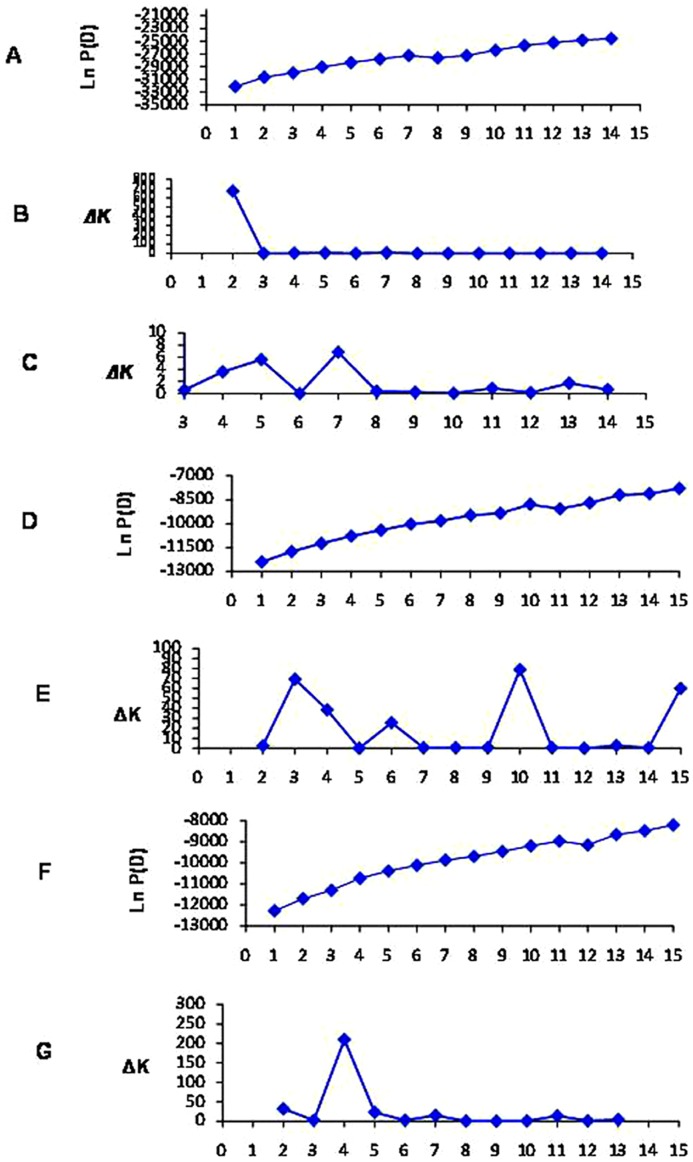
The average LnP(D) and ΔK in the total panel and inferred groups. **A–C** LnP(D) with k = 1–15, *ΔK* with k = 2–15, and *ΔK* with k = 3–15 for simulations using all 158 accessions; **D–E** LnP(D) with k = 1–15 and ΔK with k = 2–15 for inferred G1 group; **F–G** LnP(D) with k = 1–15 and *ΔK* with k = 2–15 for inferred G2 group.

**Figure 3:**
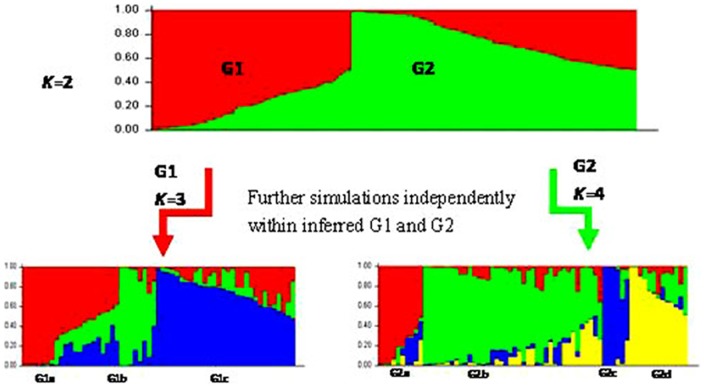
Relationship between the inferred populations. The two inferred clusters (k = 2) resulted from simulation using all 158 accessions in one and correspond to G1 and G2, respectively. Then three and four clusters (k = 3 and 4) were inferred within inferred G1 and G2 independently.

Population structure inference for the panel of 158 cotton accessions was performed by using a model-based software STRUCTURE and 212 molecular markers. The structure analysis was performed by setting the number of clusters (*K*) from 1 to 15 with five replications for each *K*. The LnP(D) value increased continuously with *K* from 1 to 15 (Figure 2A), and the highest *ΔK* value was observed at *K* = 2 followed by a drastic decline of *ΔK* from *K* = 3 (Figure 2B). Accordingly, the total panel could be assigned into two main groups, designated as G1 and G2, respectively. Using a probability of membership threshold of 60%, 58 lines were assigned to G1, 73 lines to G2 and the remaining 27 lines were considered as intermediates (Figure 3, Table S3). G1 is consisted of 19 American variety accessions, 34 Chinese variety accessions, 3 Former Soviet Union variety accessions and 2 Africa variety accessions. G2 is consisted of 15 American variety accessions, 55 Chinese variety accessions, 1 Africa variety accessions, 1 Pakistan variety accession and 1 Former Soviet Union variety accession. The intermediates are consisted of 8 American variety accessions, 17 Chinese variety accessions, 1 Auatralia variety accession and 1 French variety accession. The two groups inferred from structure did not show an association with the geographic origin of the materials, reflecting the probable extensive exchange of parental lines by breeders worldwide. The tree-based analyses gave very similar results as the STRUCTURE analysis (Figure S1).

The continuous increase of LnP(D) after *K* = 2 and a small peak of *ΔK* at *K* = 7 (Figure 2C) implied there are subtle sub-structures within the two inferred groups. Therefore, we performed further independent simulations within each of the two groups. For the G1 group, two sharp peaks of *ΔK* appeared at *K* = 3 and *K* = 10, respectively (Figure 2E), but most lines were assigned to intermediates (membership probabilities less than 60%) at *K* = 10. Accordingly, *K* = 3 was regarded as ideal number of cluster for the G1 group. For the G2 group, a sharp peaks of *ΔK* appeared at *K* = 4, implying four subgroups were included (Figure 2G). The G1 group was classified into three subgroups, named as G1a, G1b and G1c, and a mixed subgroup including 13 lines. G1a contained 12 lines, 8 of which were breeding lines from China, and the remaining 4 lines were from USA. G1b contained 7 lines, which were representative of five lines collected from north early maturity cotton area in China. G1c contained 26 lines, most of which collected from abroad (America, Former Soviet Union, and Africa). The G2 group included four subgroups, named as G2a, G2b, G2c, and G2d, and a mixed subgroup including 11 lines. G2a contained 10 lines, 4 of which were lines from China, and the remaining 6 lines were from USA. G2b contained 36 lines, which originated from USA(3), Africa(1), and China(32). G2c contained 5 lines, among which there were four innovative lines created by Atomic energy mutation of one America-originated breeding line, Arcot-1. G2d contained 11 lines, which were representative of Xingtai79–11, a breeding line from Huanghe River valley area in China and Sulian8908, a breeding line from Former Soviet Union (Figure 3, Table S1, Table S4).

**Figure 4:**
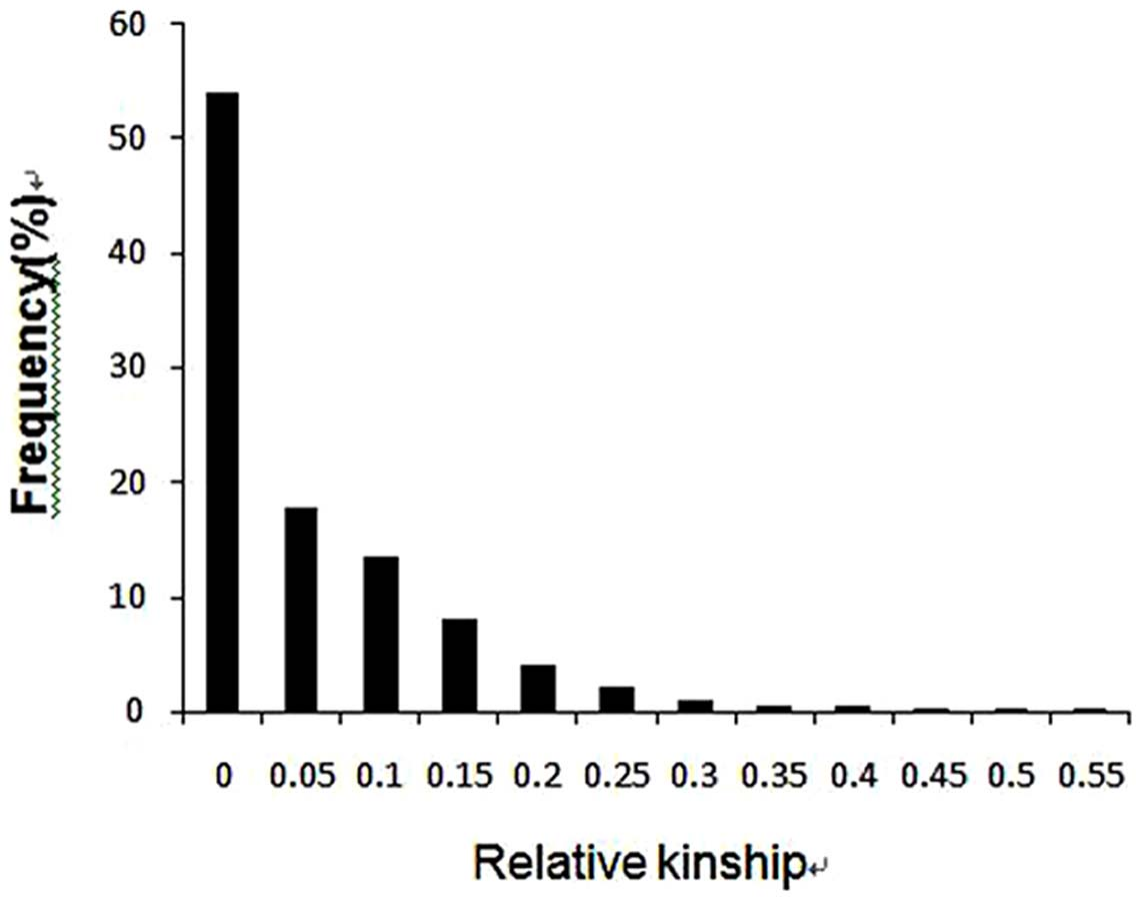
Distribution of pairwise relative kinship estimates between 158 cotton accessions. Values are from SPAGeDi estimates using 212 SSRs. For simplicity, only percentages of relative kinship estimates ranging from 0 to 0.50 are shown.

The pairwise kinship estimates based on 212 informative molecular markers showed that the majority of the pairs of cotton accessions(53.67%) had zero estimated kinship values, while 18.34% kinship estimates ranged from 0 to 0.05, 13.51% from 0.05 to 0.1, and 10.96% of the pairs had a value 0.1–0.20. The remaining pairs of accessions (3.52%) had >0.25 kinship values, suggesting involvement of some common parental genotypes in the breeding history of these germplasm groups (Figure 4). These results indicated that most lines in the panel have no or very weak kinship, which might be attributed to the broad range collection of genotypes and the exclusion of similar genotypes before analysis.

### Population differentiation

The genetic diversity within and among predefined groups (G1a–c,G2a–d) was estimated by AMOVA test. AMOVA results indicated that 4.46% (*P*<0.05) of the total molecular variation in the panel was attributed to the differentiation between groups, and 17.08% (*P*<0.01) was attributed to the differentiation among subgroups (Table 2). Although differentiation among subgroups was highly significant (P<0.0001), 76.95% of total genetic variance was attributed to the difference within subgroups and 1.51% within individuals. Pairwise *F*st of the two inferred groups was 0.10320 (P<0.001), suggesting that G1 is significantly divergent from G2. The levels of differentiation between subgroups were variable with *F*st ranging from 0.15315 (G2a with G2b, P<0.001) to 0.57518 (G1b with G2c, P<0.001) (Table 3). The differentiation among subgroups in the G1 group was lower than that in the G2 group. Among subgroups in the G2 subgroup, the differentiation between G2c with G2d was the highest (Fst = 0.40944, P<0.001) while the difference between G2a and G2b was the lowest (Fst = 0.15315, P<0.001).

**Table 2:** Analysis of molecular variance (AMOVA) among inferred populations.

Source of variation	df	Sum of squares	Variance components	Percentage of variation	P-value
Among groups^a^	1	398.569	1.3784	4.46	0.03715±0.00557
Among populations^b^	5	889.309	5.27178	17.08	<0.0001
Within populations	96	4605.758	23.75531	76.95	<0.0001
Within individuals	103	48	0.46602	1.51	<0.0001
Total	205	5941.636	30.87151		

a Groups were defined by two inferred groups.

b Populations were defined by inferred subgroups.

**Table 3:** Fst among seven subgroups.

Groups	Subgroups	G1			G2			
		G1a	G1b	G1c	G2a	G2b	G2c	G2d
G1	G1a							
	G1b	0.32395**						
	G1c	0.15490**	0.20997**					
G2	G2a	0.24737**	0.39020**	0.19033**				
	G2b	0.17797**	0.28545**	0.15914**	0.15315**			
	G2c	0.38511**	0.57518**	0.33934**	0.35653**	0.28013**		
	G2d	0.27666**	0.42016**	0.25889**	0.24459**	0.17229**	0.40944**	

** Significant at P<0.001.

### Genetic diversity within groups and subgroups

The allele number, allelic richness, gene diversity and PIC were calculated to estimate the genetic diversity in the total panel, each inferred group and subgroup. The total number of alleles in the total panel was 480, with an average of 2.26 alleles per locus. The gene diversity, PIC, and allele richness were 0.34 (0.0126–0.7159), 0.28 (0.0125–0.6643), and 2.26 (2.00–4.00), respectively (Table 4). The G1 and G2 groups contained 465 and 447 alleles, with 2.18 and 2.09 alleles per locus, respectively. Though the sample size of G2 was larger than that of G1, G1 had a higher level of gene diversity (z = −5.260, P<0.001), PIC (z = −5.484, P<0.001) and allele richness (z = −2.910, P = 0.004). Within G1, subgroup G1c and G1b contained the highest (average of 2.08) and lowest (average of 1.57) number of alleles per locus, respectively. Within G2, the number of alleles in each G2 subgroup ranged from 285 (G2c, 1.34 alleles per locus) to 418 (G2b, 1.97 alleles per locus). Group-specific alleles, gene diversity, PIC and allelic richness showed a similar trend in subgroups (Table 4).

**Table 4:** Summary of genetic diversity for overall panel, groups and subgroups.

		G1					G2						
Items	overall	G1 overall	G1a	G1b	G1c	G1 mixed	G2 overall	G2a	G2b	G2c	G2d	G2 mixed	Mixed
sample size	158	58	12	7	26	13	73	10	36	5	11	11	27
Alleles	480	465	385	333	441	423	447	368	418	285	353	377	443
Alleles per locus	2.26	2.19	1.82	1.57	2.08	2	2.11	1.74	1.97	1.34	1.67	1.78	2.09
Gene diversity	0.34	0.35	0.27	0.19	0.34	0.33	0.3	0.24	0.28	0.12	0.19	0.26	0.33
PIC	0.28	0.28	0.22	0.16	0.28	0.27	0.24	0.2	0.23	0.09	0.16	0.21	0.27
Allelic richness	2.26	2.19	1.82	1.57	2.08	2	2.11	1.74	1.97	1.34	1.67	1.78	2.09
Group-specific alleles	480	18	4	1	12	5	6	1	2	1	0	3	4

**Note:** Groups G1 and G2 were classified based on the results of STRUCTURE analysis of the 158 cotton lines.

The G1 group were further partitioned into G1a, G1b and G1c subgroups, and the G2 group into G2a, G2b, G2c and G2d subgroups.

The intermediates in the total panel, G1 group and G2 group were named as “Mixed”, “G1 mixed” and “G2 mixed”, respectively.

### Pairwise linkage disequilibrium and LD decay in the whole genome level

As the 158 cotton accessions could be divided into two distinct groups or seven subgroups, pairwise LD estimates were performed in the total panel and in each group and subgroup using a total of 212 molecular markers. In the total panel, the average *r* ^2^ of global marker pairs was 0.0132, and only 1.83% of the total possible marker locus pairs were in significant LD (*P<*0.001), suggesting that the LD level is very low in the panel (Table 5). Moreover, the average *r* ^2^ of linked marker pairs was 0.0362, and the percentage of linked marker pairs in significant LD (*P<*0.001) was 8.33%, both of which were higher than those for unlinked marker pairs (0.0121 and 0.972%, respectively), demonstrating that physical linkage is predominant in determining LD compared with random forces [51]. For global marker pairs, the mean *r* ^2^ both in groups (ranging from 0.0213 to 0.0267) and in subgroups (ranging from 0.0354 to 0.1379) was larger than that in the total panel, suggesting that the LD level was elevated when the panel was classified into groups and subgroups (Table 5). Further analysis of the LD in all groups and subgroups showed that both average *r* ^2^ and proportion of significant LD for linked markers were still higher than those for unlinked markers, which reinforced the view that physical linkage strongly influences LD in this panel of inbred lines.

**Table 5:** LD in the entire panel, groups and subgroups at the whole genome level.

	Global^c^		Unlinked^d^		Linked^e^	
Groups^a^	*r* ^2^	Significant LD (%)^f^	*r* ^2^	Significant LD (%)^f^	*r* ^2^	Significant LD (%)^f^
G1 overall^a^	0.0267	0.76	0.0257	0.58	0.0469	4.47
G1a^b^	0.1379	0.05	0.1365	0.03	0.1706	0.41
G1c	0.0506	0.27	0.0498	0.17	0.067	2.22
G2 overall	0.0213	0.94	0.0202	0.71	0.0459	6.47
G2b	0.0354	0.35	0.0343	0.21	0.0593	3.34
Total	0.0132	1.83	0.0121	0.972	0.0362	8.33

a Groups G1 and G2 were classified based on the results of STRUCTURE analysis of the 158 cotton lines.

b The G1 group were further partitioned into G1a, G1b and G1c subgroups, and the G2 group into G2a, G2b, G2c and G2d subgroups. But the G1b, G2a, G2c and G2d subgroups were not included in the analysis due to their small population size.

c The whole set of marker pairs, including linked and unlinked markers pairs.

d Pairs of markers from different chromosomes.

e Pairs of markers on the same chromosome.

f Significant threshold is set to P<0.001, which determine whether pairwise LD estimate is significant statistically.

To explore the LD at the single chromosome level, we performed the same evaluations for the single chromosome as those for the whole genome, using SSR markers evenly distributing in the single chromosome. In this part, only five chromosomes (Chr.11, 16, 18, 19 and 23) were choosed for analyzing, which representing the concentrated distribution of Verticillium wilt resistance related QTLs reported by previous studies [8]–[11]. In order to get enough marker pairs for LD estimation at a single chromosome level, we set the significance of *r* ^2^ value with P<0.05. In the total panel, the percentage of mean locus pairs in significant LD was 19.25% ranging from 14.29% to 26.46%, and mean *r* ^2^ was 0.03165 ranging from 0.0221 to 0.0442. The Chr.16 showed a relative higher mean *r* ^2^ values and highest percentage of linked marker pairs in significant LD in these five chromosomes. What's more, both in the group G1 and G2, we detected the highest mean *r* ^2^ values (0.0494 and 0.0613, respectively) and most SSR locus pairs in significant LD (8.13% and 15.83% of marker pairs, respectively) in Chr.16 in the five chromosomes, implying the stronger linkage in this chromosome. At the individual chromosome level, for Chr.11, 18 and 23, the G1 group had more SSR locus pairs in significant LD and higher mean *r* ^2^ values than the G2 group. While for Chr.16, the G1 group had less SSR locus pairs in significant LD and lower mean *r* ^2^ values than the G2 group (Table 6).

**Table 6:** LD in the entire panel, groups and subgroups at single chromosome level.

Chr.	No. of loci	Overall^a^		G1^c^						G2			
				Overall		G1a^d^		G1c		Overall		G2b	
		*r^2^*	Significant(%)^b^	*r^2^*	Significant(%)	*r^2^*	Significant(%)	*r^2^*	Significant(%)	*r^2^*	Significant(%)	*r^2^*	Significant(%)
11	21	0.0442	16.5	0.0461	12.42	0.1241	3.64	0.0643	7.5	0.043	9.09	0.0546	9.26
16	25	0.0362	26.46	0.0494	16.27	0.1582	5.88	0.0699	6.88	0.0613	25.83	0.0581	11.67
18	9	0.02212	14.29	0.0465	25	0.1807	4.76	0.0901	11.54	0.0208	20	0.0439	6.67
19	24	0.0286	22.5	0.0319	9.8	0.1613	12.62	0.0581	3.95	0.0327	6.67	0.0554	8.57
23	17	0.02712	16.48	0.0471	16.48	0.1277	3.85	0.0779	6.06	0.0365	15.38	0.0499	10
Mean	19.2	0.031648	19.246	0.0442	15.994	0.1504	6.15	0.07206	7.186	0.03886	15.394	0.05238	9.234

a The total panel for the 158 cotton lines.

b Significant threshold is set to P<0.05, which determine whether pairwise LD estimate is significant statistically.

c Groups G1 and G2 were classified based on the results of STRUCTURE analysis of the 158 cotton lines.

d The G1 group were further partitioned into G1a, G1b and G1c subgroups, and the G2 group into G2a, G2b, G2c and G2d subgroups. But the G1b, G2a, G2c and G2d subgroups were not included in the analysis due to their small population size.

Triangle plots for pairwise LD between SSR markers revealed significant LD block, or so called a genome-wide LD decay in the genome-wide LD analysis (Figure S2). However, we only estimated the LD decay distances on Chr.11, 16, 19 and 23 because of the similar informative marker coverage on these chromosomes (Table 7). In this study, *r* ^2^ threshold of 0.1 was adopted according to previous study [52]. We found that for the same gene pool, different chromosomes showed a very big change in LD decay distances. For example, in the total panel, LD decay distance was 1–2 cM for Chr.16 and 19, but 5–10 cM for Chr.23 and 15–20 cM for Chr.11. In the G1 group, LD decay distance was 10–15 cM for Chr.19 and 20–25 cM for Chr.23, but >50 cM for Chr.16 and >100 cM for Chr.11. At the same time, for the same chromosome, different gene pools showed different LD decay distance. For each of the four chromosomes, the total panel had faster LD decay than each of the two groups. A much slower decay of LD within groups might be attributed to the limited population size and narrow genetic background that inhibit LD decay [53]. These general descriptions of LD decay distance provide important information concerning decisions on marker densities for future association analyses at the chromosome level.

**Table 7:** Average LD decay distance(cM) in different chromosomes in the total panel, G1 and G2 groups for locus pairs with *r* ^2^>0.1 at P<0.05.

Chr.	Overall^a^	G1	G2
11	15–20	>100	—b
16	1–2	>50	40–50
19	1–2	10–15	—
23	5–10	20–25	10–15

a The total panel for the 158 cotton lines

b The short horizontal line means that only a few marker pairs were in significant LD that a regression curve was not created to estimate the LD decay.

### Marker loci associated with Verticillium wilt resistance

Associations between 212 marker loci and Verticillium wilt resistance were determined by GLM method. The Significant (P<0.05) candidate markers associated with disease resistance were detected in the two environments (Table 8). A total of 42 marker loci with the R^2^ ranged of 2.84–10.93% were identified to be significantly associated with Verticillium wilt resistance in at least one environment, six each on chromosome (Chr.) 11 and 16, four on Chr.19, three each on Chr.8, 17 and 23, two each on Chr.5 and 20, one each on Chr.1, 3, 4, 9, 15, 21 and 26, and the other six without location and position information. Of them, 15 and 32 markers were significantly associated with the RDI from the greenhouse environment and the disease nursery environment, respectively. Five markers, including NAU3828 on Chr.5, DPL0222 on Chr.9, BNL1606 on Chr.17, NAU3574 on Chr.20 and BNL3649 on Chr.21, were significant for both environments. Ten marker loci, significantly associated with Verticillium wilt resistance, including NAU3828 on Chr.5 at 24.1 cM, NAU3201 on Chr.8 at 72 cM, BNL3255 on Chr.8 at 76.5 cM, BNL2441 on Chr.16 at 76.567 cM, NAU2627 on Chr.16 at 62.301 cM, BNL3319 on Chr.16 at 57.702 cM, NAU2887 on Chr.16 at 60.867 cM, NAU5120 on Chr.16 at 47.7 cM, JESPR274 on Chr.23 at 52.972 cM and NAU1047 on Chr.23 at 97.1 cM, were found to be consistent with previously identified association of the marker loci from QTL mapping analyses[8], [10], [11]. The remaining SSRs are new unreported markers revealing associations with Verticillium wilt resistance in this set of cotton germplasm.

**Table 8:** Marker loci significantly associated with Verticillium wilt resistance and their positions on chromosomes (Chr).

			greenhouse		disease nursery	
Marker name	Chr.	Position(cM)	P value^a^	Rsq_Marker^b^	P value	Rsq_Marker
BNL2599	1	1.633	0.0221	0.0597	NS	
NAU5233	3	108	NS		0.034	0.0287
NAU3592	4	119.269			0.0057	0.0488
NAU3828	5	24.1	0.0282	0.0387	6.89E-04	0.0727
NAU3212	5	66	NS		0.0441	0.0531
BNL3255	8	76.5	0.0224	0.0411	NS	
NAU3201	8	38.4	0.0113	0.0524	NS	
NAU3499	8	65.3	0.0037	0.0871	NS	
DPL0222	9	137.829	0.0339	0.0371	0.0014	0.0665
NAU3074	11	183.689	NS		0.0308	0.0303
CIR196	11	145.826	NS		0.0064	0.0684
NAU980	11	169.5	NS		0.0078	0.045
NAU5428	11	32.076	NS		6.86E-04	0.1093
BNL1034	11	184.577	NS		0.0017	0.0805
NAU5064	11	162.6	NS		0.0143	0.0543
BNL2646	15	48.8	NS		0.0067	0.0463
BNL2441	16	76.567	NS		0.0017	0.067
NAU2627	16	62.301	0.0349	0.037	NS	
BNL3319	16	57.702	NS		0.0011	0.067
TMB1114	16	41.815	NS		8.39E-04	0.0695
NAU2887	16	60.867	NS		5.66E-04	0.0755
NAU5120	16	47.7	NS		0.0209	0.0341
BNL1606	17	51.762	0.0101	0.0526	0.0098	0.0427
NAU2859	17	86.286	NS		0.0184	0.065
JESPR101	17	71.031	NS		4.50E-04	0.0956
BNL4069	19	36.8	0.009	0.0619	NS	
JESPR0001	19	123.567	0.0477	0.0479	NS	
CIR364	19	66.663	NS		0.0037	0.0708
NAU2894	19	26.581	NS		0.0184	0.0357
BNL3646	20	3.479	0.0465	0.0317	NS	
NAU3574	20	58.598	0.0411	0.0506	0.0443	0.0399
BNL3649	21	10.8	0.0076	0.0566	0.0338	0.0291
NAU2954	23	114.846	0.0093	0.0542	NS	
JESPR274	23	52.972	NS		0.017	0.0874
NAU1047	23	97.1	NS		0.0173	0.0362
NAU4912	26		0.0226	0.0594	NS	
NAU5463	—	—	NS		0.009	0.0432
Gh268	—	—	NS		0.0066	0.0466
Gh454	—	—	NS		0.006	0.0509
NAU3563	—	—	NS		0.0353	0.0284
w11330	—	—	NS		0.0065	0.065
73686–3	—	—	NS		0.0313	0.0461

a NS, not statistically significant;

b Rsq_marker, total explained phenotypic variation.

## Discussion

### Genetic diversity in the cotton panel

In this study, across the entire population, we observed an average number of alleles per locus of 2.26 which ranged from 2 to 4, a gene diversity of 0.34 and a PIC of 0.28 (Table 4). These values were similar to those detected in 53 *Gossypium hirsutum* L. cotton cultivars [54] and 8 cotton (*G. hirsutum*) cultivars [55], reflecting a relatively low genetic diversity in *Gossypium hirsutum* L. cotton cultivars worldwide. However, the average number of alleles per locus, gene diversity and PIC of our study are less than those detected in 47 accessions including 38 *G. hirsutum*, 2 *G. darwinii*, 2 *G. tomentosum* and 5 *G. barbadense* [56] and 35 cultivars and eight inbred lines of *G. hirsutum* L. from Africa, United States and Brazil [57]. Although the number of alleles in this study was lower than that detected in 97 cultivars and primitive species [58], the PIC values in the two studies were very similar. This can be explained by that the level of polymorphism among races and wild species of *Gossypium* was significantly higher than that within cultivated *G.hirsutum*, and cultivars domesticated directly in a native cotton growing area usually reserved their higher level of polymorphism than those in a non-native cotton growing area.

Though the genetic diversity is relatively low in the entire panel, the group G1 and G2 showed a significant differences in gene diversity and PIC. When compared with G2, G1 had a higher level of gene diversity and PIC. Significant differentiation assessed in AMOVA was observed between the two groups. G1 group has 18 group-specific alleles while G2 group 6 group-specific alleles (Table 4). The reason might be due to that the G1 group in this study contained more lines from abroad than G2. In the subgroup level, we detected a a significant difference of allele richness between China originated lines (G1b) and abroad originated lines (G1c). This can be explained by the fact that China is not a native cotton growing area, and most cotton varieties planted in China were derived from a few sources of germplasm such as DPL, Stoneville, King, Uganda, Foster, and Trice, all of which were introduced from abroad [59].

### Population structure and differentiation in the association panel

Detailed knowledge about population structure in an association panel is important to avoid spurious associations. A model-based approach using the software STRUCTURE[44], [60] might be the most frequently used method to correct spurious associations. It is computationally difficult to obtain accurate estimates of the number of populations(*K*). Generally, *K* is taken to be the value with the highest estimated LnP(D) value returned by STRUCTURE [44]. However, in real data the value of LnP(D) continues to increase with increasing K. In this situation, an ad hoc measure *ΔK* based on the relative rate of change in the likelihood of the data between successive *K* values were used to determine the optimal number of clusters [45]. In this study, the *ΔK* values indicated dividing the cotton panel into two groups and seven subgroups was the most biologically meaningful population structure. Our results are very similar to a cluster analysis for 53 *Gossypium hirsutum* L. cotton cultivars, which were grouped into two large groups and seven subgroups [54]. And 35 cultivars and eight inbred lines of *G. hirsutum* L. also were identified as four groups that consisted of American cultivars and inbred lines, African and Brazilian cultivars, BRS Brazilian cultivars and FM Brazilian cultivars by a structure running [57]. Also, 285 exotic *Gossypium hirsutum* accessions were classified into three groups consisted of landrace stock germplasm group, Mexican varieties and African varieties and 334 *G. hirsutum* variety accessions were identified as three groups consisted of Uzbekistan, Latin American, and Australian cotton accessions in Uzbek cotton germplasm collection [29], [28]. These results demonstrated the existence of population structure in cotton germplasm of *G. hirsutum* worldwide.

Several studies had showed that the genetic structure of *G. hirsutum* L. is in accordance with their geographical origins [55], [28], [29], [57]. But in our study, it was interesting to note that in each of the two groups there were germplasm lines from several origins (China, America, Africa and former Soviet Union), indicating the exchange and domestication of germplasm between these origins. In the level of subgroups, we only detected a limited association between the subgroup structure and the geographic origin of the materials, for example, all the lines in G1b originated from China, and most lines in G2c containing the pedigree of Arcot-1, one America-originated breeding line. But other subgroups consisted of accessions derived both China and abroad. So, most of the elite *G. hirsutum* variety accessions in China had the close pedigree relationships with some exotic variety accessions (especially accessions derived from America). This result was consistent with the report by Cheng and Du (2006), who considered that many of the Chinese breeding source germplasms had been based on the introduction, selection, and domestication of germplasms from other countries thus narrowing their genetic base and possibly making them vulnerable to the present and future diseases [59]. Iqbal et al. (2001) also pointed out that the *G. hirsutum* cultivated around the world is derived from the USA, which were exported to other countries in the 19th and early twentieth century, with most upland cotton used in early Chinese cotton breeding coming from this source [61]. Though the apparent lack of diversity in cultivated *G. hirsutum*, Van Esbroeck and Bowman (1998) have argued that there is enough allelic variation, mutation or recombination in crosses between closely related individuals to allow improvement in agronomic performance and/or that the coefficient of parentage may not reflect the real genetic distance [62]. In our study we observed that all the accessions in the subgroup G2c had good fiber qualities with a fiber length of >30 mm and a fiber strength of >30 cN/tex (unpublished), which would effectively improve the fiber quality by justifying crosses between these accessions and other related individuals in cotton cultivar breeding programs.

Our current results showed the significant differentiation among groups and subgroups in our association panel. Pairwise Fst showed that G1group is significantly divergent from G2 with the fact that G1 contained more lines from abroad and had more group-specific alleles than G2. The highest differentiation between subgroups occurred in the G1b with G2c. This can be explained by all the lines in G1b originated from China and most of lines in G2c owned the pedigree of Arcot-1, one America-originated breeding line, implying the highly differentiation between China-originated and America-originated breeding lines. In G1, pairwise Fst values indicate that G1b was strongly differentiated from G1a and G1c. In G2, pairwise Fst values indicated that G2c was highly unrelated with G2a, G2b and G2d. These results suggested that subgroup having a single origin or pedigree was usually apt to differentiate with those having a mixed origin. A few lines in some subgroups were not consistent with pedigree information perfectly, maybe due to the unknown pedigree information in our study.

### Patterns of linkage disequilibrium in the cotton panel

The genome-wide distribution of LD estimated with a high number of markers greatly influence the resolution of association mapping [63]. In this study, we observed that a total of 8.33%, 4.47%, and 6.47% of the linked loci pairs in the entire panel, G1 group and G2 group, respectively, showed significant LD (P<0.001) (Table 5). The percentages observed in our study were lower than those reported earlier [28], [29]. This can be explained by that different significance thresholds and different plant materials were used in these studies.

Physical linkage that determines LD between molecular marker and causative polymorphisms is the genetic basis for association mapping of genes or QTLs underlying traits of interest [16]. In this study, the extent of LD of linked markers in the entire panel, groups and subgroups is significantly higher than that of unlinked markers (Table 5), suggesting that physical linkage strongly influences LD in this cotton panel, and indicating that this cotton panel is suitable for association analysis. Triangle plots for pairwise LD between SSR markers revealed significant LD decay in the genome-wide LD analysis (Figure S2). As a supplementary, we analyzed the LD delay distance in the cotton panel in a whole genome scale (Figure S3), and found that the LD in the cotton panel decayed to the background level within 10–15 cM in a whole genome scale. If we set the r^2^ threshold of 0.2, genome-wide LD fast decayed within 1–3 cM (Figure S3). Therefore, in our association panel, the LD decayed faster than that in 335 variety accessions of *G. hirsutum* from Uzbek cotton germplasm collection, which indicated that a genome-wide average of LD extended up to genetic distance of 25 cM at r^2^≥0.1 and reduced to ∼5–6 cM at r^2^≥0.2 [28]. This can be explained by that the genetic diversity in our association panel (overall PIC for SSRs was in the range of 0.0125–0.06643 with an average of 0.28) was higher than that in 335 variety accessions of *G. hirsutum* from Uzbek cotton germplasm collection (overall PIC for SSRs was in the range of 0.006–0.50 with an average of 0.082). On the other hand, the LD in our panel delayed slower than that in 208 landrace stocks of G. *hirsutum*, which indicated that LD clearly decays within the genetic distance of <10 cM with r^2^≥0.1 and reduced to ∼1–2 cM at r2≥0.2 [29]. This difference can be explained by that landraces, usually having a higher genetic diversity, often showed faster LD decay than modern varieties [64].

Population structure is one of several important factors that have strong influences on LD [16]. In our LD estimations, we took into account the effect of population structure by subdividing the total panel into different groups and subgroups. Various levels of LD in groups and subgroups were observed, indicating that population structure has significant impact on LD (Table 5). Based on LD analyses both in the whole genome level and at the individual chromosome level, the LD level was elevated when the panel was classified into groups and subgroups (Table 5 and Table 6), implying that variable extents of LD are expected within the different genetic groups and highlight the fact that different marker densities will be required if association studies are planned in the different genetic groups.

So far, there is no report about the LD at the single chromosome level in cotton. Our results showed that for the same gene pool, different chromosomes showed a notable change in LD decay distances. In the total panel, Chr.11 and 23 showed wider LD decay distance than Chr.16 and 19, indicating that Chr.11 and 23 may carry more QTLs or genes related to important agronomic traits that were strongly selected in breeding [65]. In G1 and G2 gene pools, the Chr.16 showed higher mean r^2^ values and wider LD decay distance than other chromosomes, implying the stronger linkage in this chromosome. In fact, both our study and previous study [11] proved the existence of QTL clusters for Verticillium wilt resistanc on Chr.16, which was consistent with the strong linkage in this chromosome.

### Verticillium wilt resistance associated markers and QTL identification

In this study, two hundred and twelve genome-wide distributed markers were employed in the association study (Table S2). Of more than 60 previously identified Verticillium wilt resistance QTLs [8]–[11], only ten were confirmed to be consistent with them. Unlike previous study in bi-parental populations [11], which showed that 41QTLs related to Verticillium wilt resistance intensively distributed on chromosomes D9(Chr.23) and D7(Chr.16), our study identified 42 associations widely distributed on 15 chromosomes(Table 8).This implied that association mapping can locate many QTLs over the entire genome since the mapping population includes a large number of diversified entries of germplasm, while conventional QTL mapping based on bi-parental populations only identified fewer QTLs which be located in a limited area in the genome where the two parents differ, thus causing QTL clustering [11].

On Chr.1, 3, 4, 9, 15, 21and 26, we identified one Verticillium wilt resistance associated marker from each chromosome. These marker loci were regarded as novel Verticillium wilt resistance QTLs that have not been reported, and it was the first findings for Chr.1, 3, 4, 9, 15 and 21 to exist Verticillium wilt resistance related QTLs on them. What's more, DPL0222 on Chr.9 and BNL3649 on Chr.21 were considered stable Verticillium wilt resistance QTLs that showed significant association with Verticillium wilt resistance both in the greenhouse environment and in the disease nursery environment.

On Chr.5 we identified two markers, NAU3212 and NAU3828. NAU3212 was located at 66 cM according to Guo et al.[33] and was regarded as a novel Verticillium wilt resistance QTL that has not been reported. NAU3828 was located at 24.1 cM and overlapped with the Verticillium wilt resistance QTL *qVL-A5-2BC_1_S_2_592* found by Yang et al. [8]. What's more, this marker showed significant association at P<0.05 level in the greenhouse environment and strong association at P<0.0001 level in the disease nursery environment. So, the marker NAU3828 was regarded as a stable QTL in different environments and co-localized with previously identified Verticillium wilt resistance QTLs.

On Chr.8 our study identified NAU3201, NAU3499 and BNL3255. NAU3201 located at 38.4 cM and overlapped with the Verticillium wilt resistance QTL *qVL-A8-1F_2_* found by Yang et al. [8]. BNL3255 at 76.5 cM had been reported near the QTL *qVV-A8-1BC_1_S_2_BP2* between NAU3964 at 70.7 cM and NAU920 at 129.1 cM [8]. NAU3499 at 65.3 cM was a novel Verticillium wilt resistance QTL that has not been reported.

On Chr.11 we identified five significant marker-trait associations, which were different from three loci having large effect on resistance to Verticillium wilt reported by Bolek et al.[9]. Of them, NAU5428 showed strong association with Verticillium wilt resistance at P<0.0001 level and explained the most phenotypic variation (10.93%), thus might be a new major QTL for Verticillium wilt resistance that need to be further identified.

On Chr.16, a report indicated that there existed QTL clusters with high contribution rates for Verticillium wilt resistance on this chromosome [11]. Of six identified markers in our study, TMB1114 was a novel Verticillium wilt resistance QTL that has not been reported, and other five were deduced to be co-localized with previously identified Verticillium wilt resistance QTLs. BNL2441 at 76.567 cM was overlapped with q8.24-2 reported by Wang et al.[10] and close to the QTL *qV-BP2M-D7-1* (58.5∼72.2) by Jiang et al.[11]. NAU2627 at 62.301 cM was within the QTL *qV-BP2M-D7-1* (58.5∼72.2 cM) and *qV-VD8M-D7-2* (60.9∼67.2 cM) reported by Jiang et al. [11]. BNL3319 at 57.702 cM was within the QTL *qV-VD8M-D7-1* (52.9∼60.9 cM) and close to the QTL *qV-BP2M-D7-1* (58.5∼72.2 cM) reported by Jiang et al. [11]. NAU2887 at 60.867 cM was within the QTL *qV-BP2M-D7-1* (58.5∼72.2 cM) and *qV-VD8M-D7-1* (52.9∼60.9 cM), and close to the QTL *qV-VD8M-D7-2* (60.9∼67.2 cM). NAU5120 at 47.7 was within the QTL *qV-BP2S1-D7-1* (39.9∼50.1) and *qV-T9M-D7-1* (42.5∼71.9) reported by Jiang et al. [11], and close to the *qV-VD8M-D7-1* (52.9∼60.9 cM). Therefore, our study further proved the QTL clusters on Chr.16. These QTL clusters confirmed by multiple studies strongly suggests a reliable location harboring Verticillium wilt resistance QTL.

On Chr.23, three markers NAU2954, JESPR274 and NAU1047 were identified. NAU2954 was regarded as a novel Verticillium wilt resistance QTL that has not been reported. JESPR274 at 52.972 cM, explaining relatively high phenotypic variation (8.74%), was close to the QTL *qV-VD8S2-D9-3* (47.5∼51.5) reported by Jiang et al. [11]. NAU1047 was one of the flanking markers for each of *qV-T9S1-D9-1, qV-MIXS2-D9-1* and *qV-MIXM-D9-3* reported by Jiang et al. [11].

The remain identified markers in our study included three on Chr.17, four on Chr.19, two on Chr.20, and six without location and position information. All these marker loci were considered novel Verticillium wilt resistance QTLs that have not been reported. Of them, BNL1606 on Chr.17 and NAU3574 on Chr.20 were considered stable QTLs that showed significant association with Verticillium wilt resistance both in the greenhouse environment and in the disease nursery environment.

Totally, in our study, ten SSR markers were colocalized with or close to previously identified Verticillium wilt resistance QTLs using conventional QTL mapping approaches. This suggests that association mapping using natural population can effectively detect major QTLs. Moreover, most SSR loci (32 of 42) were considered novel Verticillium wilt resistance QTLs that have not been reported, implying that association mapping has the advantage of being able to work with a higher number of polymorphic markers than conventional QTL mapping and locates many QTLs over the entire genome.

## Conclusion

Two groups and seven subgroups were identified in the cotton panel, demonstrating the existence of population structure in cotton germplasm of *G. hirsutum* worldwide. The two subgroups inferred from structure did not show an association with the geographic origin of the materials, reflecting the probable extensive exchange of parental lines by breeders worldwide. In the subgroup level, subgroup having a single origin or pedigree was usually apt to differentiate with those having a mixed origin. This fact suggested that it is a prerequisite to perform structure analysis before association mapping. Both in the whole genome level and at the individual chromosome level, the LD level was elevated when the panel was classified into groups and subgroups, highlighting the fact that different marker densities will be required if association studies are planned in the different genetic groups. For the same gene pool, different chromosomes showed a very big change in LD decay distances, indicating that different chromosomes may carry different QTLs or genes related to important agronomic traits that were strongly selected in breeding. Association mapping based on the disease nursery and greenhouse environment identified 42 marker loci associated with Verticillium wilt resistance, which widely distributed on 15 chromosomes, implying that association mapping can locate many QTLs over the entire genome. 10 marker loci were found to be consistent with previously identified QTLs, which suggests that association mapping using natural population can effectively detect major QTLs. 32 loci were new unreported markers related with Verticillium wilt resistance, implying that association mapping has the advantage of being able to work with a higher number of polymorphic markers than conventional QTL mapping and locates many QTLs over the entire genome. QTL clusters for Verticillium wilt resistanc on Chr.16 were proved by our study, which was consistant with the strong linkage in this chromosome.

## Supporting Information

Figure S1 Unrooted neighbor-joining tree for 158 accessions. The ancestries of the accessions in inferred populations are represented by different colours.
(TIF)

Figure S2 The triangle LD plot for a pairwise genome-wide LD between SSR loci (with a 5% minor allele filtered datasets). Polymorphic SSR sites are plotted on both X-axis and Y-axis. Each cell represents the comparison of two pairs of SSR sites with the color codes for the presence of significant LD. Colored bare code for the significance threshold levels is given.
(TIF)

Figure S3 LD decays (*r* ^2^) in the association panel consisting 158 cotton lines. The *r* ^2^ value for marker distance of 0 cM is defined as 1. The dots are *r* ^2^ values for linked marker pairs in significant LD (*P*<0.001). The curve was drawn across the dots using the nonlinear regression model. The horizontal line indicates the thresholds of *r* ^2^ = 0.1 and *r* ^2^ = 0.2, respectively.
(TIF)

Table S1 Accesion or cultivar, origin, subspecies, type and usage of the materials, and groups of 158 accessions. The varieties were sorted according to their STRUCTURE membership probability as Figure 3. ^a^ means that the innovation line was created by interspecific hybridization; ^b^ means that the innovation line was created by induced mutagenesis; ^c^ Subgroups defined by STRUCTURE.
(XLS)

Table S2 Microsatellite markers used in the study, including the location of the markers in the cotton reference map and position based on the position references. ^a^ The chromosome locations were based on the the AD-genome wide Reference Map. ^b^ The positon information was based on position references. ^c^ Position references for the information of the SSR: A. Yu JZ; Kohel RJ; Fang DD; Cho J; Van Deynze A; Ulloa M; Hoffman SM; Pepper E; Stelly DM; Jenkins JN; Saha S;Kumpatla SP; Shah MR; Hugie WV; Percy RG. A High-Density Simple Sequence Repeat and Single Nucleotide Polymorphism Genetic Map of the Tetraploid Cotton Genome. G3: Genes, Genomes, Genetics Mission 2012 2(3): 43–58. B. Guo W; Cai C; Wang C; Han Z; Song X; Wang K; Niu X; Lu K; Shi B; Zhang T. A microsatellite-based, gene-rich linkage map reveals genome structure, function and evolution in gossypium. Genetics 2007 176(1): 527–541. C. Yu Y; Yuan D; Liang S; Li X; Wang X; Lin Z; Zhang X. Genome structure of cotton revealed by a genome-wide SSR genetic map constructed from a BC1 population between gossypium hirsutum and G. barbadense. BMC Genomics 2011 12:15. D. Shen X; Guo W; Lu Q; Zhu X; Yuan Y; Zhang T. Genetic mapping of quantitative trait loci for fiber quality and yield trait by RIL approach in Upland cotton. Euphytica 2007 155 371–380. E. Lacape JM, Jacobs J, Arioli T, Derijcker R, Forestier-Chiron N, Llewellyn D, Jean J, Thomas E, Viot C. A new interspecific, Gossypium hirsutum x G. barbadense, RIL population: towards a unified consensus linkage map of tetraploid cotton.Theor Appl Genet. 2009 119(2):281–92. F. Yu JW; Yu SX; Lu CR; Wang W; Fan SL; Song MZ; Lin ZX; Zhang XL; Zhang JF; Wu W. High-density Linkage Map of Cultivated Allotetraploid Cotton Based on SSR, TRAP, SRAP and AFLP Markers. Journal of Integrative Plant Biology. 2007 49: 716–724. H. Qin H; Guo W; Zhang YM; Zhang T. QTL mapping of yield and fiber traits based on a four-way cross population in Gossypium hirsutum L. Theor Appl Genet 2008 117:883–894. N. Ma XX; Zhou BL; L YH; Guo WZ; Zhang TZ. Simple Sequence Repeat Genetic Linkage Maps of A-genome Diploid Cotton (Gossypium arboreum). Journal of Integrative Plant Biology 2008 50: 491–502. P. Wang HM; Lin ZX; Zhang XL; Chen W; Guo XP; Nie YC; Li YH. Mapping and Quantitative Trait Loci Analysis of Verticillium Wilt Resistance Genes in Cotton. Journal of Integrative Plant Biology. 2007 50:174–182. R. Guo WZ; Cai CP; Wang CB; Zhao L; Wang L; Zhang TZ. A preliminary analysis of genome structure and composition in Gossypium hirsutum. BMC Genomics 2008 9: 314. — The locus was not assigned to the chromosomes of known maps. The SSR marker E38644, W11330, 73686–3 and 6738–1 were EST-SSRs developed from sequences by RNA-Seq for a cotton sample innoculated with Verticillium dahlia (unpublished). ^e^ The marker 1DF and 1EF are RGAP markers.
(XLSX)

Table S3 Proportional memberships in groups as defined by Structure.
(XLSX)

Table S4 List of accessions with their proportional memberships in model-based subgroups.
(XLSX)
