# Supplementary figures and images for: Genetic Structure, Linkage Disequilibrium and Association Mapping of Verticillium Wilt Resistance in Elite Cotton (Gossypium hirsutum L.) Germplasm Population
Source: PLoS One. 2014 Jan 23;9(1):e86308. doi: 10.1371/journal.pone.0086308 (PMC3900507; doi:10.1371/journal.pone.0086308)

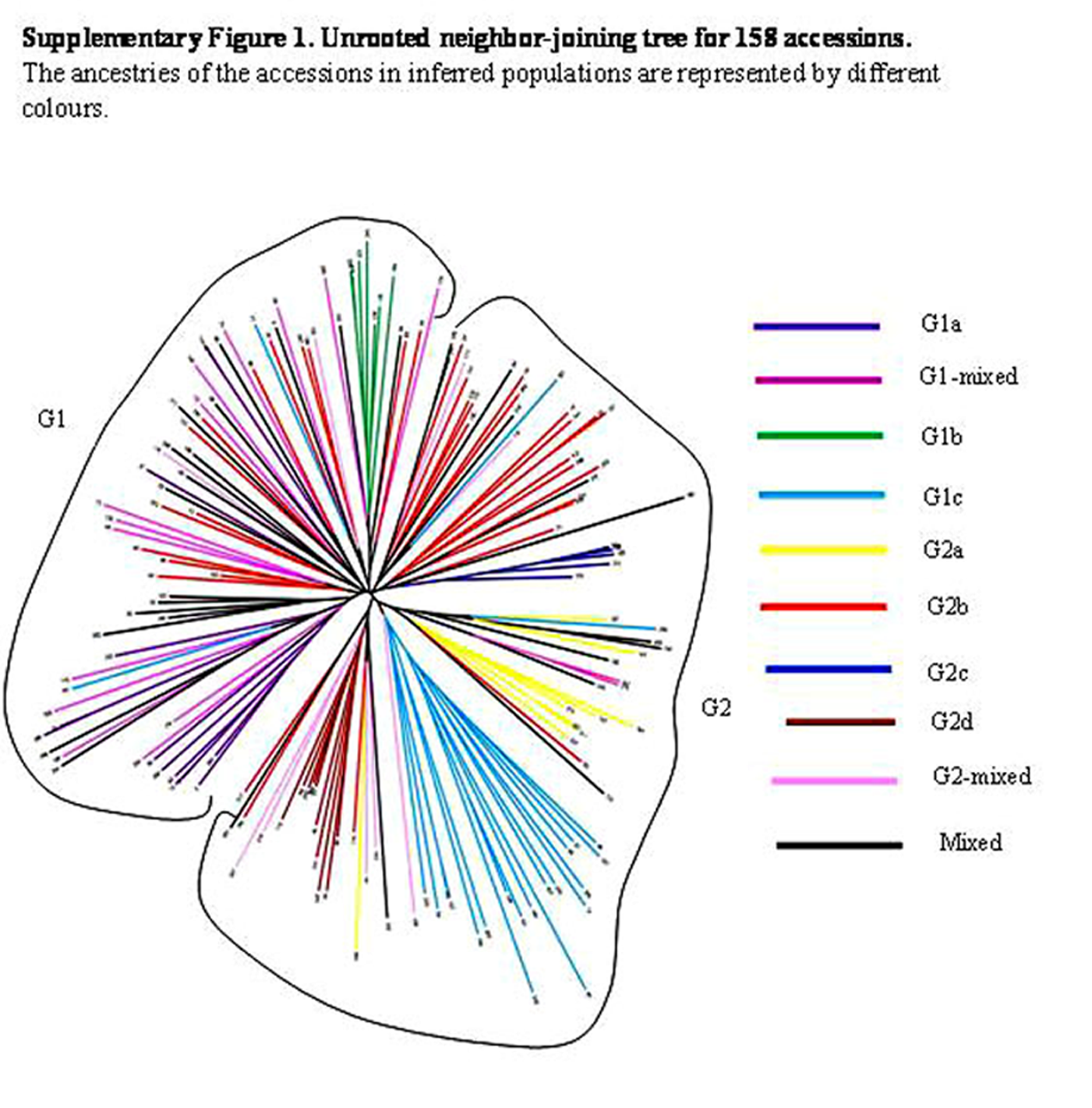

Supplement: Figure S1 — Unrooted neighbor-joining tree for 158 accessions. The ancestries of the accessions in inferred populations are represented by different colours. (TIF) [file pone.0086308.s001.tif]

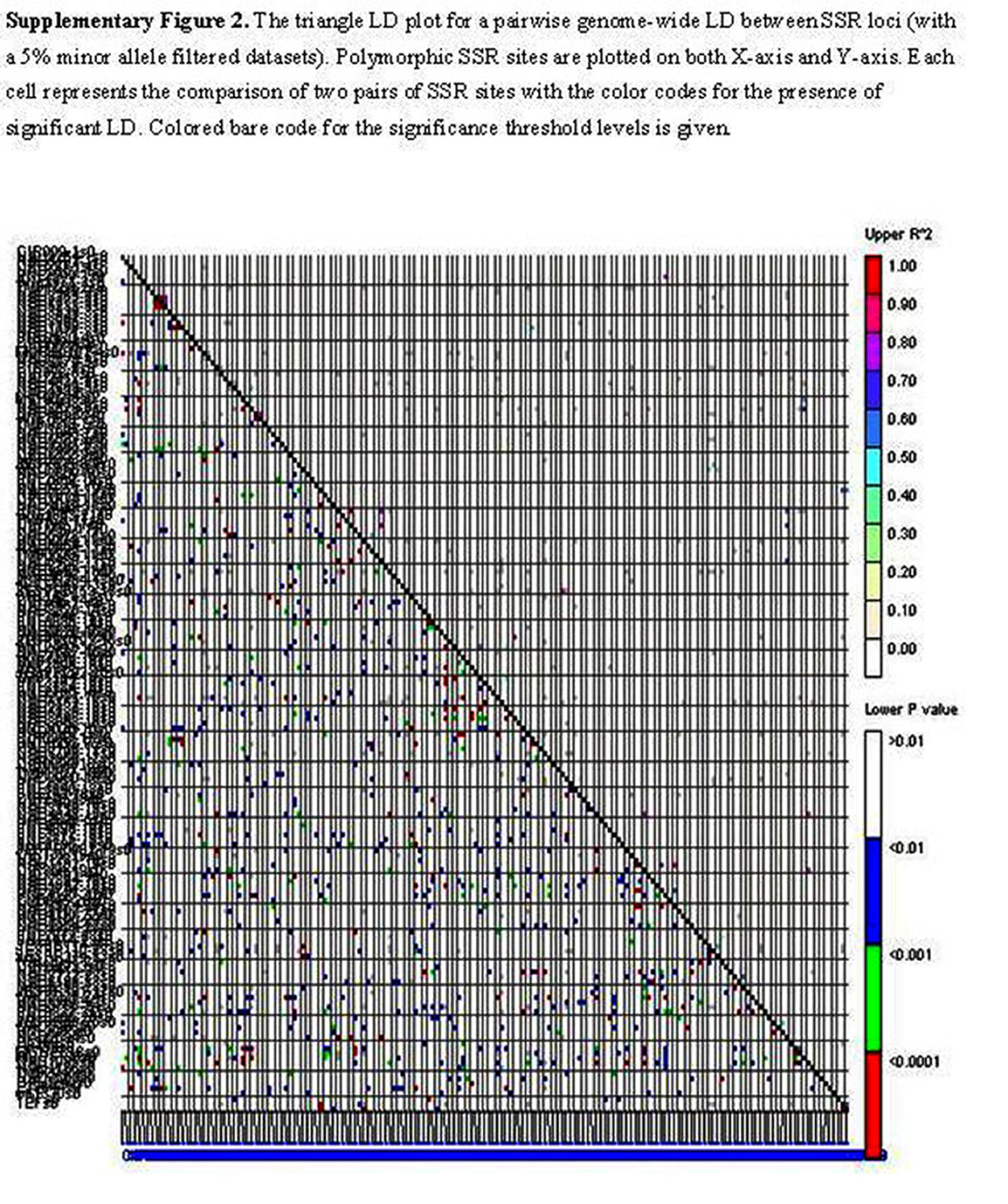

Supplement: Figure S2 — The triangle LD plot for a pairwise genome-wide LD between SSR loci (with a 5% minor allele filtered datasets). Polymorphic SSR sites are plotted on both X-axis and Y-axis. Each cell represents the comparison of two pairs of SSR sites with the color codes for the presence of significant LD. Colored bare code for the significance threshold levels is given. (TIF) [file pone.0086308.s002.tif]

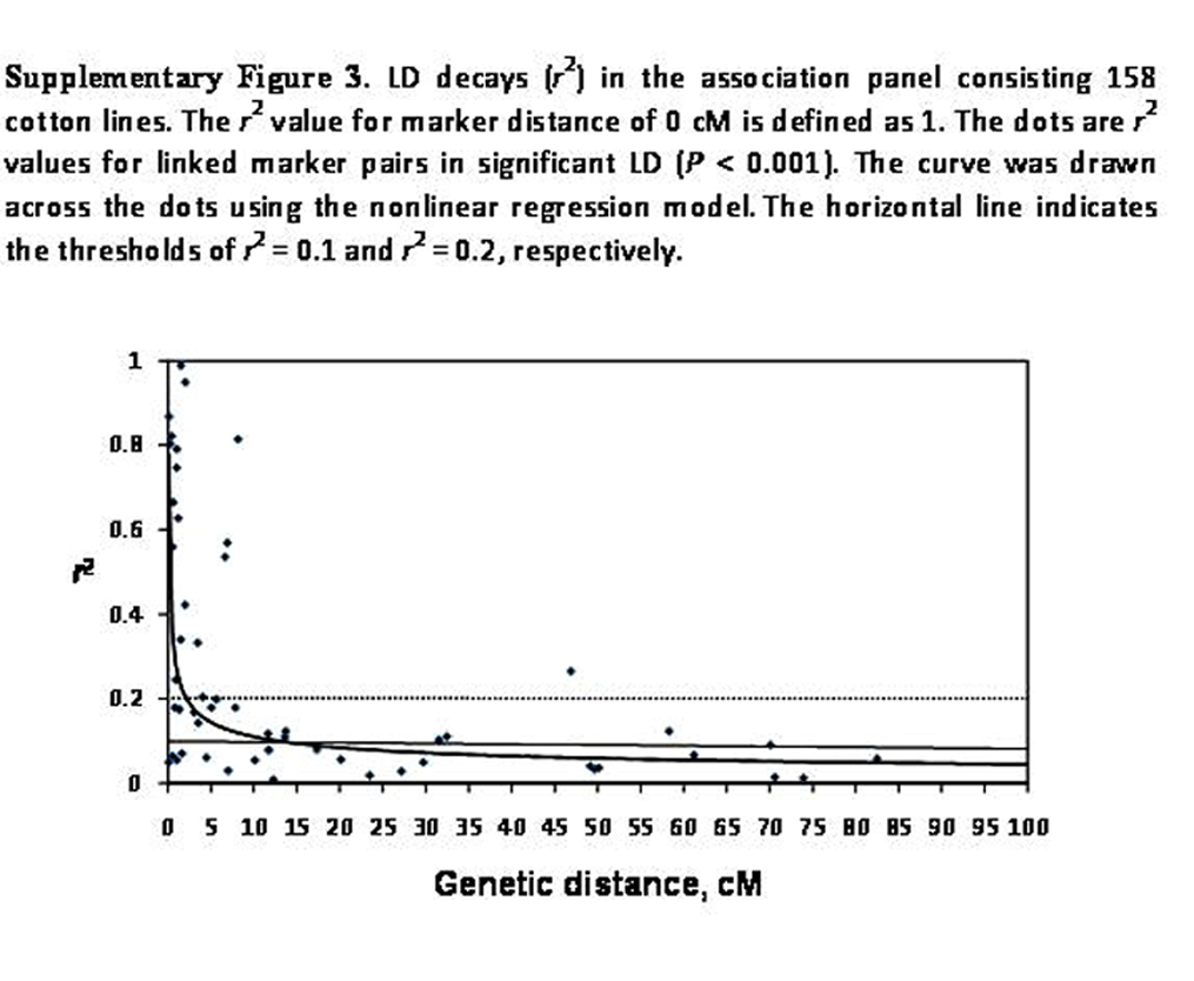

Supplement: Figure S3 — LD decays (r 2) in the association panel consisting 158 cotton lines. The r 2 value for marker distance of 0 cM is defined as 1. The dots are r 2 values for linked marker pairs in significant LD (P<0.001). The curve was drawn across the dots using the nonlinear regression model. The horizontal line indicates the thresholds of r 2 = 0.1 and r 2 = 0.2, respectively. (TIF) [file pone.0086308.s003.tif]
